# Evolutionary algorithm search for network connectivities conducive to periodic behavior at sub-spiking frequencies

**DOI:** 10.1186/1471-2202-14-S1-P393

**Published:** 2013-07-08

**Authors:** Daniel T Robb, Natalia Toporikova

**Affiliations:** 1 Department of Mathematics, Computer Science and Physics, Roanoke College, Salem, VA 24153, USA; 2 Department of Biology, Washington and Lee University, Lexington, VA 24450, USA

## 

We use an evolutionary algorithm (EA) to search the space of leaky integrate-and-fire (LIF) neuron networks, in order to identify network connectivities producing significant rhythmic activity at sub-spiking frequencies (i.e., 'bursting-like behavior'). We find that the connectivities of the most-fit LIF networks exhibit a relatively broad in-degree distribution and a relatively narrow out-degree distribution. We examine the frequencies of connection motifs in the most-fit LIF networks as compared to random networks. In a network of more realistically modeled neurons, the most-fit network connectivities are observed to produce a broader frequency response as compared to that resulting from random network connectivities.

**Figure 1:**
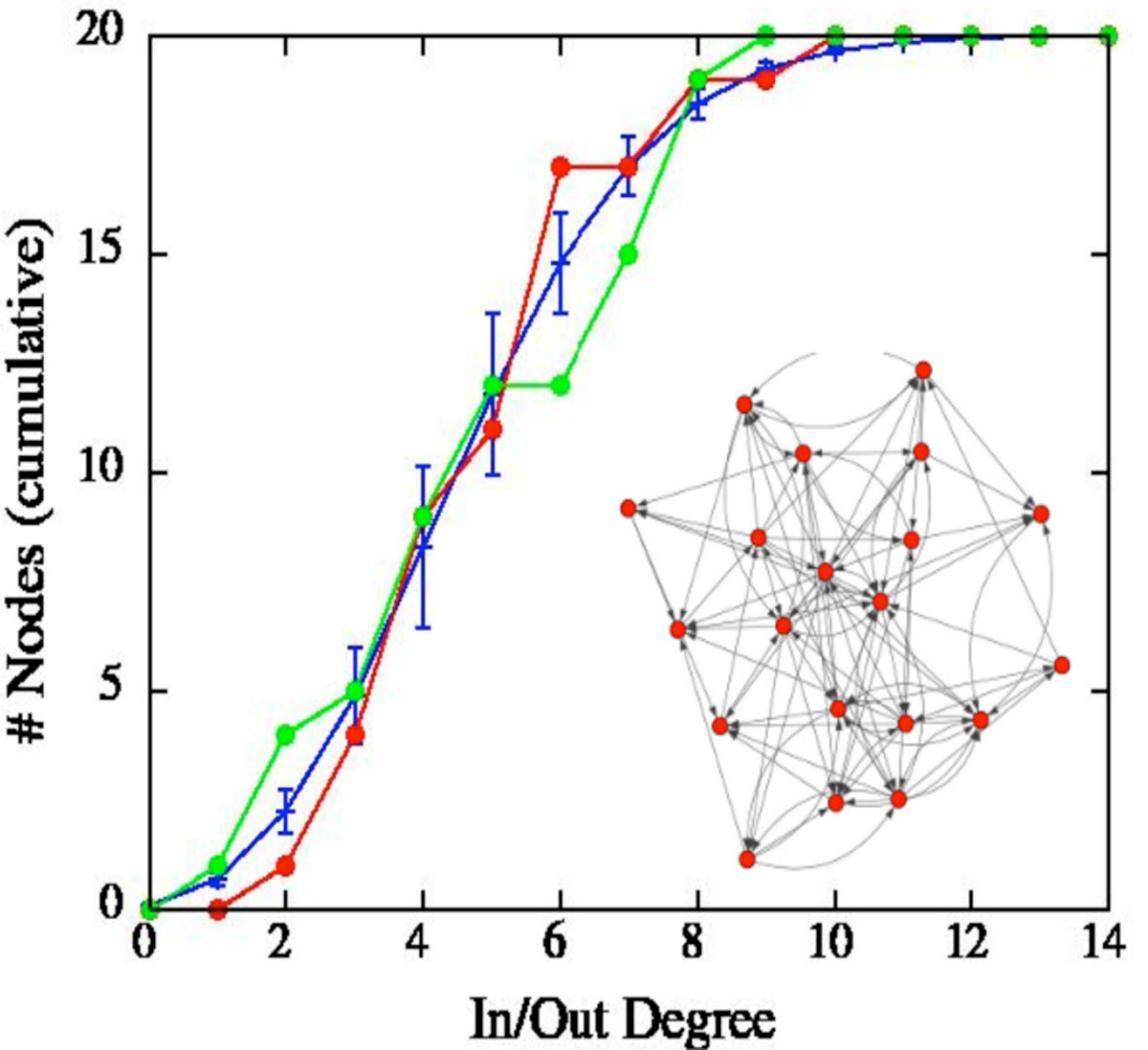
**shows the cumulative in- and out-degree distributions of a representative most-fit network, whose connectivity graph is illustrated in the inset**. The orange line represents the cumulative in-degree distribution, and the red line the cumulative out-degree distribution. The blue bars represent the expected cumulative distribution (and error bars) for random network connectivities.

Figure 1 shows the cumulative in- and out-degree distributions of a representative most-fit network, whose connectivity graph is illustrated in the inset. The orange line represents the cumulative in-degree distribution, and the red line the cumulative out-degree distribution. The blue bars represent the expected cumulative distribution (and error bars) for random network connectivities.

